# Intracellular Environment Improvement of *Mycobacterium neoaurum* for Enhancing Androst-1,4-Diene-3,17-Dione Production by Manipulating NADH and Reactive Oxygen Species Levels

**DOI:** 10.3390/molecules24213841

**Published:** 2019-10-25

**Authors:** Minglong Shao, Youxi Zhao, Yu Liu, Taowei Yang, Meijuan Xu, Xian Zhang, Zhiming Rao

**Affiliations:** 1 The Key Laboratory of Industrial Biotechnology of Ministry of Education, School of Biotechnology, Jiangnan University, Wuxi 214122, China; mlshao@jiangnan.edu.cn (M.S.); ytw1228@163.com (T.Y.); xumeijuan@jiangnan.edu.cn (M.X.); zxshengwu@126.com (X.Z.); 2 Beijing Key Laboratory of Biomass Waste Resource Utilization, College of Biochemical Engineering, Beijing Union University, Beijing 10023, China; zhaoyouxi@buu.edu.cn

**Keywords:** androst-1,4-diene-3,17-dione, intracellular environment, NADH oxidase, catalase, *Mycobacterium neoaurum*

## Abstract

As one of the most significant steroid hormone precursors, androst-1,4-diene-3,17-dione (ADD) could be used to synthesize many valuable hormone drugs. The microbial transformation of sterols to ADD has received extensive attention in recent years. In a previous study, *Mycobacterium neoaurum* JC-12 was isolated and converted sterols to the major product, ADD. In this work, we enhanced ADD yield by improving the cell intracellular environment. First, we introduced a nicotinamide adenine dinucleotide (NADH) oxidase from *Bacillus subtilis* to balance the intracellular NAD^+^ availability in order to strengthen the ADD yield. Then, the catalase gene from *M. neoaurum* was also over-expressed to simultaneously scavenge the generated H_2_O_2_ and eliminate its toxic effects on cell growth and sterol transformation. Finally, using a 5 L fermentor, the recombinant strain JC-12*_yodC-katA_* produced 9.66 g/L ADD, which increased by 80% when compared with the parent strain. This work shows a promising way to increase the sterol transformation efficiency by regulating the intracellular environment.

## 1. Introduction

As one of the well-known androgen steroids, androst-1,4-diene-3,17-dione (ADD) was extensively used as an important precursor for the synthesis of steroid hormone medicines in the pharmaceutical industry [1]. Traditionally, ADD was obtained from natural steroids such as sapogenin and diosgenin using multistep chemical degradation and modification methods. However, the well-established route of sapogenin and diosgenin to ADD has many drawbacks, such as waste of land resources, high-cost processes, relatively low yields, and high pollution [2]. With the awareness of environmental protection, biological technology has become the development tendency and inevitable choice for the steroid medical industry [3,4].

Since the discovery of microbial sterols side-chain degradation to 17-ketosteroids, sterol biotransformation has become a promising alternative way to synthesize valuable steroid intermediates in the pharmaceutical industry [5]. Among all the microorganisms that could degrade sterols to steroids, *Mycobacterium* was known as the most promising ADD producing strain [6]. Owing to the distinguished work of finding sterol metabolism gene clusters in *Mycobacterium* [7], many works have focused on the identification and characterization of these enzymes recently [8,9,10]. There have also been some efforts made to improve sterol biotransformation. Wei et al. over-expressed 3-ketosteroid-Δ^1^-dehydrogenase (KSDD) in *M. neoaurum* to increase soybean phytosterol bioconversion [11]. Su et al. used cofactor engineering to maintain the balance of redox to promote steroid biotransformation [12]. In our previous study, we used stepwise pathway engineering to strengthen the metabolic flux of the sterols for the improvement of ADD production [13]. However, few articles reported about the intracellular environment of *Mycobacterium*, which is important for strain growth and sterol biotransformation.

The cell intracellular environment mainly contains adenosine diphosphate (ADP), adenosine triphosphate (ATP), nicotinamide adenine dinucleotides (NADH and NAD^+^), nicotinamide adenine dinucleotide phosphate (NADPH and NADP^+^) and reactive oxygen species (ROS) [14]. NADH and NAD^+^ play important roles in cell physiological activities and participate in almost all of the metabolic pathways in industrial strains [15,16,17]. As it was postulated, the bioconversion equation of 1 mol β-sitosterol to ADD was shown: β-sitosterol + 21 NAD^+^ + 10 FAD + 4 ATP +7 P_i_ + 7 GDP + 21 H_2_O = ADD + 21 NADH + 10 FADH_2_ + 4 AMP + 7 GTP + 4 PP_i_ + 21/2CO_2_ + 21 H^+^ [12,18]. Therefore, the NADH/NAD^+^ regeneration and the maintenance of the redox balance are considered as the rate-limiting factors in the steroid synthetic pathway and important factors for the steady state of the cell intracellular environment [12]. As the toxic intermediates for the cell intracellular environment, ROS, including hydrogen peroxide (H_2_O_2_) and hydroxyl radicals (∙OH), are produced due to the incomplete oxidation during aerobic metabolism [14]. Additionally, H_2_O_2_ generated during the flavin adenine dinucleotide (FAD) regeneration was reported in our previous study [19]. Thus, it is important to decrease the high level of ROS and to maintain the balance of the cell intracellular environment during steroid synthesis.

**Figure 1:**
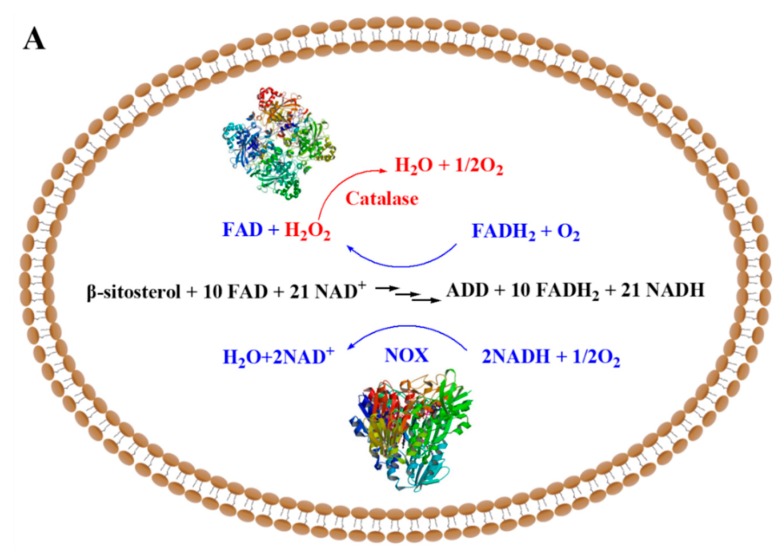

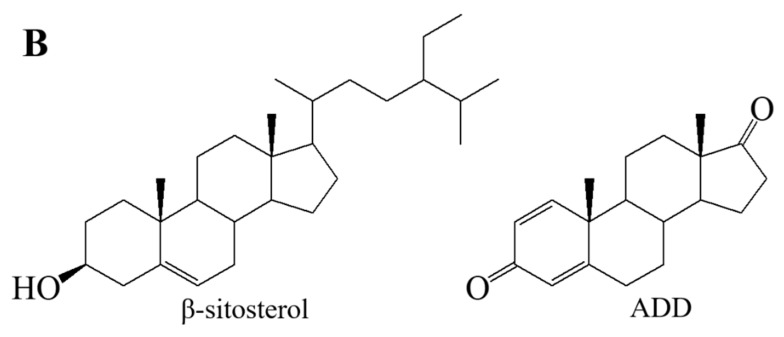
The NOX and catalase were co-expressed to rebalance the cell intracellular environment during biotransformation of sterols to androst-1,4-diene-3,17-dione (ADD) in *Mycobacterium neoaurum*. (**A**) sterol biotransformation to ADD; (**B**) structural formula of β-sitosterol and ADD.

*M. neoaurum* JC-12 converting phytosterol to ADD was obtained by mutation in our lab [20]. In this study, we maintained the balance of the cell intracellular environment in *M. neoaurum* JC-12 to enhance phytosterol conversion efficiency. First, the NADH oxidase (NOX) from *Bacillus subtilis* [21], was expressed in *M. neoaurum* JC-12 to construct the intracellular NAD^+^ regeneration. Then, catalase catalyzing dismutation of H_2_O_2_ into H_2_O and O_2_, was expressed to eliminate any toxic effects caused by high ROS level (Figure 1). Finally, the recombinant strain JC-*12_yodC-katA_* produced 9.66 g/L ADD on a 5 L bioreactor, which is 1.8-fold of the production by parent strain JC-12. This study supplies new insight into maintaining the balance of the cell intracellular environment to improve the production of steroid precursors by sterol biotransformation.

## 2. Results and Discussion

### 2.1. The Increase in NAD^+^ Availability by Expressing NOX Resulted in an Improved ADD Yield in the NAD^+^ Regeneration System of JC-12_yodc_

During the phytosterol bioconversion pathway, the intracellular NAD^+^ concentration and availability decreased as the NAD^+^ was largely consumed. Construction of the NAD^+^ regeneration system by over-expressing NOX in engineered JC-12*_yodC_* could enhance the intracellular NAD^+^ pool and further strengthen the sterol metabolic flux. The successful construction of the recombinant strain JC-12*_yodC_* was verified by plasmid extraction and gene sequencing.

**Figure 2:**
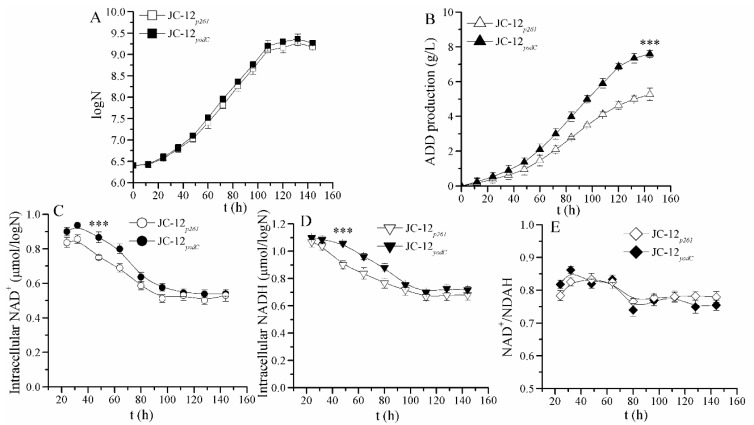
Time profiles of ADD fermentation and intracellular NADH and NAD^+^ concentrations by strain JC-12*_p261_* (hollow) and strain JC-12*_yodC_* (solid). (**A**) the cell growth; (**B**) the ADD production, (**C**) intracellular NAD^+^ concentration; (**D**) intracellular NADH concentration; (**E**) intracellular NAD^+^/NADH ratio. N, the number of CFU (colony forming units) per mL of culture broth. An amount of 20 g/L phytosterol was used as a substrate to carry out the fermentation. The results are shown in biological triplicate. One-way ANOVA was used to examine the mean differences between the points of the data groups. *** *p* < 0.001. The statistical significance has been found between the two analyzed strains. Error bars show standard deviations.

As shown in Figure 2A, no difference in biomass was observed between JC-12*_p261_* and JC-12*_yodC_*, indicating that *M. neoaurum* cell growth was not affected by NOX expression. However, recombinant JC-12*_yodC_* produced 7.53 g/L ADD, which increased by 43% when compared with JC-12*_p261_* (5.26 g/L) at 144 h (Figure 2B). This result indicates that the functional NOX expression resulted in an increased NAD^+^ availability, which further improved the NAD^+^-dependent sterols catabolism flux. To explain this phenomenon, the NADH and NAD^+^ intracellular concentrations were determined. In these two strains, NAD^+^ and NADH intracellular concentrations continuously changed during the phytosterol transformation process (Figure 2C). In both strains, NAD^+^ and NADH concentrations decreased in cell growth phases and remained constant in non-growth phases. NOX expression in JC-12*_yodC_* resulted in a relatively higher level of NAD^+^ and NADH pools when compared with strain JC-12*_p261_* (Figure 2C,D). Meanwhile, no obvious difference in the NAD^+^/NADH ratio of these two strains was observed (Figure 2E). These results indicate that the intracellular redox balance in JC-12*_yodC_* was not disturbed, which could explain why its cell growth was not obviously affected. The “extra” NAD^+^ regenerated in JC-12*_yodC_* by NOX could be utilized in the NAD^+^ consumed pathway of sterols metabolic flux to improve ADD yield, which could explain why NOX expression has no remarkable effect on the NAD^+^/NADH ratio.

During the fermentation, the intracellular NADH and NAD^+^ concentrations are important reductants and oxidants for cellular metabolism, and they are constantly regenerated to realize redox equilibrium for continued anabolism and catabolism [16]. However, the sterol transformation pathway caused a decrease in NAD^+^ concentration and availability. In this study, we firstly introduced the NOX from *B. subtilis* to improve the NAD^+^ availability and to drive the metabolic flux of the sterol transformation pathway. As expected, the final ADD production was further improved. Similar results were shown when regulating the intracellular NADP^+^ and NADPH concentrations, the bio-production of testosterone was improved significantly [3]. By moderate-expressing NOX in *B. subtilis*, the NADH-dependent metabolic pathway was rebalanced and the acetoin production was improved [21]. Su et al. also reported that the NAD^+^/NADH ratio was an important factor and the expression of NOX could improve ADD yield [12]. This result indicates that the balance of intracellular NAD^+^ and NADH concentrations was important during the sterol transformation.

### 2.2. The Over-Expression of Catalase Eliminated the Toxic Effect of H_2_O_2_ Accumulation on Strain Growth and ADD Production

The strong oxidizer H_2_O_2_ is generated during the regeneration of flavin adenine dinucleotide (FAD) in the phytosterol transformation process. H_2_O_2_ could damage different cellular components, such as proteins, DNA and lipids [22], which may result in a potentially inhibited cell growth and ADD yield. Thus, catalase was over-expressed to increase the production of ADD by eliminating the toxic effects of H_2_O_2_. The successful construction of recombinant strain JC-12*_yodC_*_-*katA*_ was verified by plasmid extraction and gene sequencing.

In order to confirm the successful expression of NOX and catalase in recombinant strain JC-12*_yodC_*_-*katA*_, enzyme activities were analyzed and the results are shown in Table 1. Strain JC-12*_yodC_* showed the NOX activity of 337.2 mU/mg, which was about 13-fold of that of JC-12*_p261_*, while strain JC-12*_yodC_*_-*katA*_ showed the catalase activity of 235 U/mg, which was 8.7-fold of JC-12*_p261_*. Moreover, the NOX enzyme activity of JC-12*_yodC_*_-*katA*_ was similar to that of JC-12*_yodC_*, which implies that catalase expression has no effect on NOX activity. The enzymatic activity analysis showed that NOX and catalase successfully co-expressed in *M. neoaurum* JC-12.

**Figure 3:**
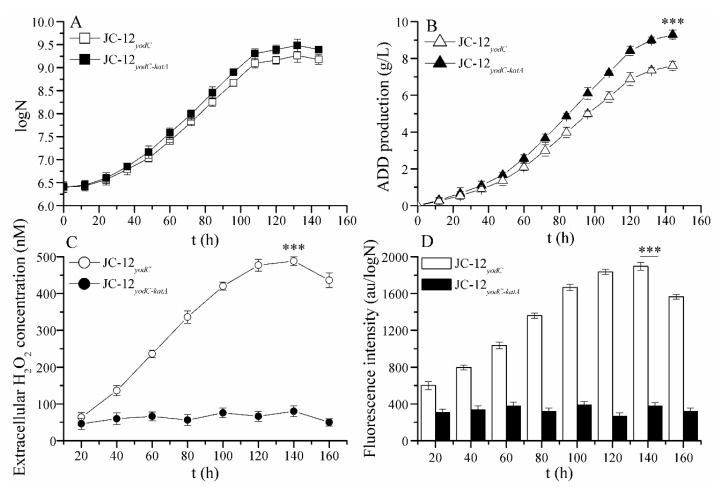
Time profiles of ADD fermentation and the extracellular H_2_O_2_ concentrations and intracellular reactive oxygen species (ROS) levels by strain JC-12*_yodC_* (hollow) and strain JC-12*_yodC-katA_* (solid). (**A**) the cell growth; (**B**) the ADD production, (**C**) extracellular H_2_O_2_ concentration; (**D**) intracellular ROS levels. N, the number of CFU (colony forming units) per mL of culture broth. 20 g/L phytosterol was used as a substrate to carry out the fermentation. The results are shown in biological triplicate. One-way ANOVA was used to examine the mean differences between the points of the data groups. *** *p* < 0.001. The statistical significance has been found between the two analyzed strains. Error bars showed standard deviations.

We also measured the extracellular H_2_O_2_ concentrations and intracellular reactive oxygen species (ROS) levels. As shown in Figure 3, compared with strain JC-12*_yodC_*_-*katA*_, the extracellular H_2_O_2_ concentrations and intracellular ROS levels of strain JC-12*_yodC_* were increased during the phytosterol conversion process, which resulted in the stagnation of both cell growth and ADD yield (Figure 3C,D). This was mainly because during the sterol conversion process, the intracellular FAD was regenerated. As a result of FAD regeneration, H_2_O_2_, which is toxic for diverse cellular components, was generated and increased. On the contrary, both the biomass and ADD production of recombinant strain JC-12*_yodC_*_-*katA*_ was higher than that of strain JC-12*_yodC_*, and the final ADD yield reached a maximum of 9.36 g/L with an increase of 24% (Figure 3A,B). This was mainly due to the fact that catalase was over-expressed in JC-12*_yodC_*_-*katA*_, which could simultaneously scavenge the generated H_2_O_2_ and eliminate its toxic effects on the diverse cellular components and sterol transformation. Therefore, over-expression of catalase was beneficial for cell growth and ADD yield.

In the sterol transformation process, H_2_O_2_ was produced within the FAD regeneration system. The accumulation of high H_2_O_2_ concentration has the potential to damage diverse cellular components and further lead to toxic effects on cell growth and ADD production. This outcome was possibly due to the lack of catalase and peroxidase activity for this strain, causing an inability to eliminate H_2_O_2_ in a timely fashion and allowing for an easy accumulation to a high concentration. High H_2_O_2_ concentration resulted in substantial damage to the proteins and DNA [23], which resulted in the inhibition of cell growth and enzyme activities. The over-expression of catalase could effectively eliminate the toxic effect of the generated H_2_O_2_, which when tested resulted in higher biomass and ADD production. Therefore, the catalase expression is needed for achieving high sterol conversion efficiency. These results indicate that the regulation of the intracellular NAD^+^/NADH and H_2_O_2_ level is a promising way to enhance the transformation efficiency of low-cost sterols to valuable steroid precursors in the pharmaceutical industry.

**Figure 4:**
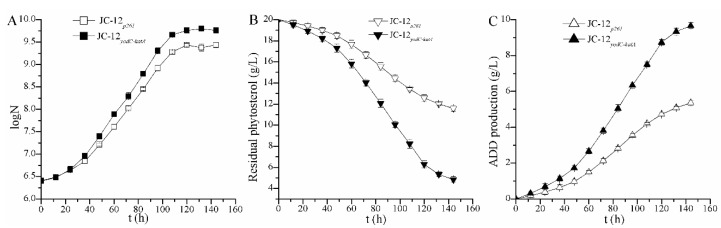
Time profiles of ADD fermentation by strain JC-12*_p261_* (hollow) and the recombinant strain JC-12*_yodC-katA_* (solid) in a 5 L fermentor. (**A**) the cell growth; (**B**) the residual phytosterol; (**C**) the ADD production. N, the number of CFU (colony forming units) per mL of culture broth. 20 g/L phytosterol was used as a substrate to carry out the fermentation. The results are shown in biological triplicate. Error bars showed standard deviations.

In order to further verify the capability of strain JC-12*_yodC_*_-*katA*_ on industry scale, a 5 L bioreactor was used to evaluate its performance with 20 g/L phytosterol. As shown in Figure 4C, the final ADD production of JC-12*_yodC_*_-*katA*_ reached 9.66 g/L at 144 h, which was 1.8-fold of the ADD production (5.36 g/L) by the original JC-12*_p261_*. All of the results confirm that the regulation of the intracellular NAD^+^/NADH and H_2_O_2_ level could be an effective way to improve sterol transformation efficiency and the production of steroid intermediates.

## 3. Materials and Methods

### 3.1. Strains and Culture Conditions

Table 2 shows the primers, plasmids and strains used in our study. Strain *E. coli* was cultured in Luria–Bertain (LB) medium and strain *M. neoaurum* was cultured in seed medium including 10 g/L glucose, 10 g/L peptone, 6 g/L beef extract, 10 g/L NaCl at pH 7.5. The fermentation medium contained 20 g/L glucose, 10 g/L peptone, 6 g/L beef extract, 3 g/L K_2_HPO_4_, 0.5 g/L MgSO_4_·7H_2_O, 5 × 10^−4^ g/L MnCl_2_·4H_2_O at pH 7.5 [13].Hydroxymethyl-β-cyclodextrin (HP-β-CD) was added to improve phytosterol solubility and the mass ratio of sterol to HP-β-CD was 1:3 (*w/w*). The fermentation was carried out in 50 mL shake flasks at pH 7.5 and 30 °C with 160 rpm agitation speed. The scale-up fermentation was carried out using a 5 L bioreactor with 400 rpm agitation speed, 1 vvm ventilation at pH 7.5 and 30 °C. Corresponding antibiotics were added when needed.

### 3.2. Over-Expression of NOX and Catalase in M. Neoaurum JC-12

We used pMV261 as an expression vector in *M. neoaurum* JC-12 to express NOX and catalase. The *yodC* gene from *B. subtilis* (Gene ID: 939506) was amplified using primers *yodC*-f/*yodC*-r and inserted to *Bam*H I/*Eco*R I sites to create recombinant plasmid pMV261-*yodC*. The strain was then transformed to obtain recombinant strain JC-12*_yodC_*. To augment the catalase expression in JC-12*_yodC_*, the *katA* gene from *M. neoaurum* (Gene ID: 674842736) was amplified by primers *katA*-SD-f/*katA*-r. The fragment of *katA* gene was then inserted into pMV261-*yodC* at *Hin*d III site to construct pMV261-*yodC*-*katA*, and the plasmid was converted into JC-12*_yodC_* to create strain JC-12*_yodC_*_-*katA*_.

### 3.3. NOX and Catalase Enzyme Activity Assays

NOX activity was determined according to a previous study [21], and one unit was defined as the amount of enzyme that produced 1 μmol of NAD^+^ per minute at pH 7.0 and 30 °C. Determination of NADH and NAD^+^ intracellular concentrations was according to the operating manual of Amplite Fluorimetric NAD^+^/NADH Ratio Assay Kit (Sunnyvale, CA, USA) [24].

Catalase activity was detected as per a previous work [19], and one unit was defined as the decomposition of 1 µmol H_2_O_2_ (ε_240_ = 43.6 × 10^3^/cm/M) per min at pH 7.0 and 30 °C. Intracellular reactive oxygen species (ROS) levels were detected using the fluorogenic probe 2’,7’-dichlorofluorescein diacetate (DCFH-DA) described previously [25,26]. Extracellular H_2_O_2_ concentrations were measured according to the operating manual of Amplex Red Hydrogen Peroxide/Peroxidase Assay Kit (Waltham, MA, USA) [14].

### 3.4. Analytical Methods

A 1 mL sample from the culture broth wasextracted with 4 mL ethyl acetate. Then, the supernatant was detected after centrifugation by high performance liquid chromatography (HPLC, Palo Alto, CA, USA) equipped with a C18 column (Diamonsil^®^C18, 5 µm particles, 250 mm × 4.6 mm) and a UV/visible detector. The mobile phase contains water and methanol of 30:70 (*v/v*) and ADD was measured at 254 nm with a column temperature of 30 °C and 1 mL/min flow rate [27]. The biomass was shown as CFU number per mL of fermentation broth during cultivation [28]. Sterol determination was carried out using gas chromatography (GC) [29].

## 4. Conclusions

In this study, in order to balance the cell intracellular environment during phytosterol transformation, we firstly introduced a water-forming NOX from *B. subtilis* to increase the NAD^+^ availability. Then, the catalase was over-expressed to eliminate the toxic effects of the H_2_O_2_ generated during the FAD regeneration system. The final ADD production using a 5 L fermentor reached 9.66 g/L with an increase of 80%. This work provides new insight to improve microbial cells for efficiently converting sterols to other valuable steroid metabolites in the pharmaceutical industry.

## Figures and Tables

**Table 1:** Specific enzyme activities of NOX and catalase in recombinant *M. neoaurum* strains.

Strains	Enzyme Activity *	
	NOX (mU/mg)	Catalase (U/mg)
JC-12*_p261_*	25.6 ± 1.5	27 ± 3
JC-12*_yodC_*	337.2 ± 9.6	23 ± 5
JC-12*_yodC_*_-*katA*_	312.3 ± 6.3	235 ± 6

Note: The results are shown in biological triplicate. * One unit of NOX enzyme activity is defined as the amount of enzyme that produced 1 μmol of NAD^+^ per minute at 30 °C and pH 7.0. One unit of catalase enzyme activity is defined as the decomposition of 1 µmol H_2_O_2_ (ε_240_ = 43.6 × 10^3^/cm/M) per min at 30 °C and pH 7.0.

**Table 2:** Primers, plasmids and strains used in this study.

Strains/Plasmids/Primers	Description	Sources
**Strains**		
*Escherichia coli*		
JM109	General host for gene cloning	Invitrogen, (Carlsbad, CA, USA)
*Mycobacterium neoaurum*		
JC-12	Wild type strain, converting sterols to ADD with small amount of AD	Lab storage, (Wuxi, China)
JC-12*_p261_*	JC-12 harboring empty plasmid pMV261	This study
JC-12*_yodC_*	NOX over-expressed strain of JC-12, harboring plasmid pMV261-*yodC*	This study
JC-12*_yodC-katA_*	Catalase over-expressed strain of JC-12*_yodC_*, harboring plasmid pMV261-*yodC*-*katA*	This study
**Plasmids**		
pMD18-T	*E. coli* clone vector; Amp^R^	Novagen, (Madison, WI, USA)
pMV261	Shuttle vector of *E. coli* and *mycobacterium*, carrying the heat shock promoter *hsp*60; Kan^R^	R. Jacobs Jr.
pMV261-*yodC*	pMV261 carrying *yodC* gene; Kan^R^	This study
pMV261-*yodC*-*katA*	pMV261-*yodC* carrying *katA* gene with its SD sequence inserted after *yodC*; Kan^R^	This study
**Primers**		
*yodC*-f	CG*GGATCC*ATGACGAATACTCTGGATG	This study
*yodC*-r	CG*GAATTC*TTACAGCCAAGTTGATAC	This study
*katA*-SD-f	ACG*AAGCTT*aagaaggagatataATGCGCGAAAGGAACAGCCC	This study
*katA*-r	ACG*AAGCTT*CTACTTGACGGCCGCCTC	This study

The restriction enzyme sites are in italics and underlined.

## Abbreviations

AD	4-androstene-3,17-dione
ADD	androst-1,4-diene-3,17-dione
CFU	colony forming units
HP-β-CD	hydroxymethyl-β-cyclodextrin
H_2_O_2_	hydrogen peroxide
ROS	reactive oxygen species
NOX	NADH oxidase
DCFH-DA	2′,7′-dichlorofluorescein diacetate
GC	gas chromatography
HPLC	high performance liquid chromatography
